# Connecting the dots: Similar visual orthographic acquisition for Braille or line junctions

**DOI:** 10.3758/s13423-025-02788-1

**Published:** 2026-02-17

**Authors:** Filippo Cerpelloni, Olivier Collignon, Hans Op de Beeck

**Affiliations:** 1 Department of Brain and Cognition, KU Leuven, Leuven Brain Institute, Leuven, Belgium; 2 Institute of Psychology (IPSY) & Institute of Neuroscience (IoNS), UCLouvain, Belgium; 3 The Sense Innovation and Research Center, HES-SO Valais-Wallis, Lausanne & Sion, Switzerland

**Keywords:** Reading, Visual perception, Word perception

## Abstract

Most written systems share basic shape features with natural objects such as line junctions, a commonality thought to be at the basis of fluent reading. Studies that compared reading acquisition for different scripts used line-based scripts and investigated them in terms of complexity, novelty, or associations of stimuli from different categories. Here, we directly compared visual Braille, a script that only includes patterns of dots and no line junctions, to Line Braille, a novel, custom-made script based on line junctions drawn between Braille dots. For four consecutive days, two groups of participants (each *N* = 40) underwent online training of either one of the scripts, during which they first mapped new letters onto the Latin alphabet and then trained on full words. Each day, participants were tested by asking them to transcribe a set of stimuli (words and pseudowords) from the novel to the Latin script. Across sessions, we found no significant differences between scripts in the overall transcription accuracy nor in the time required to transcribe words. We only found a small delay in the learning trajectory of the Braille group represented through an interaction between group and session in overall accuracy, and slightly higher sensitivity for stimulus length in the Braille group. Overall, these results show that line junctions only provide a small and temporary benefit when learning a new script, contrasting with the idea that line junctions is a core visual features for learning orthography.

## Introduction

Visual object recognition relies on the integration of visual inputs into progressively more complex objects (Logothetis & Sheinberg, 1996; Riesenhuber & Poggio, 1999, 2002). One particular case of object recognition concerns reading. Behaviourally, reading consist in the recognition of letters and words, attributing linguistic value to an arbitrary visual code. The main theories behind reading acquisition in the brain pose that regions in the ventral visual stream are co-opted to process written stimuli (Cohen et al., 2000, 2002), integrating the features that compose letters into progressively complex stimuli, culminating into words (Dehaene et al., 2005; Dehaene-Lambertz et al., 2018; Vinckier et al., 2007). Literature on this topic focused on the importance and essentiality of the lines and the junctions composing such stimuli, following studies that showed how most written scripts share the same basic properties, line-junctions, and that those properties are derived from natural objects (Changizi et al., 2006), thus hypothesising an evolution of scripts to accommodate the existing preference of the brain for the integration of line-junctions.

Prominent studies showed for example that, similarly to line drawings, disrupting the line vertices (junctions) of letters results in higher reaction times and more naming errors (Szwed et al., 2009) and that this behaviour is reflected in brain activations (Szwed et al., 2011). In contrast, several behavioural and neuroimaging studies support an alternative account of reading, focused on the connections between the visual system and language areas in the brain (Saygin et al., 2016). In particular, several scripts have been associated to VWFA activation (houses, Martin et al., 2019; faces, Moore et al., 2014; Hebrew or Korean, Baker et al., 2007; Xue & Poldrack, 2007). Putting aside the specifics of activations in VWFA, all these studies feature stimuli that, for how complex and novel they can be, are made out of lines and junctions, visual units that are common to most scripts (Changizi et al., 2006). Under this consideration, all these studies can be seen as confirmations of the adaptability of the visual reading system to complex line-based stimuli.

Little evidence is present, instead, for what happens when the line-junctions are missing. A unique case is presented by visual reading of Braille. In Braille, letters consist of a combination of one to six dots. With its uniquely different structure, it poses an interesting paradigm to study visual learning and reading acquisition. While Braille is not made of the canonical line junctions (Changizi et al., 2006), in the case of western Latin-based scripts, it maps into the same linguistic properties (orthography, phonology) of the script it mirrors. Previous work has already investigated tactile Braille reading in blind (Büchel et al., 1998; Reich et al., 2011) and sighted individuals (Gaca et al., 2025; Siuda-Krzywicka et al., 2016). However, tactile reading involves a different sensory integration pathway, and therefore the learning and the representations of words in tactile Braille in VWFA may depend on separate processes compared to visual reading. The visual reading of Braille instead relies on the same processing hierarchy that is thought to rely on the integration of line junctions (Dehaene et al., 2005). In this context, a different acquisition trajectory for visual Braille compared to a line-based script would be expected under the orthographic tuning hypothesis. Conversely, an equal performance for such scripts would indicate that the mapping between visual and language information is the predominant driver of efficient reading, independently of low-level visual features. Bola and colleagues (2017) directly compared a training on visual Braille to a training on another script, Cyrillic, leading to the conclusion that line junctions may be needed for fluent reading. Their study shows how a line-based script leads to higher accuracy and lower reaction times in a lexical decision task compared to visual Braille. This study, however, compares two groups who underwent different types of training: those who learnt Cyrillic attended a Russian language course, while those who learnt Braille were assigned with individual work to be performed in tactile Braille and checked visually. Not only the trainings were different, but visual Braille reading was not trained directly and its effect are not distinguishable from the effect of tactile Braille reading. Moreover, while Cyrillic is visually dissimilar from the Polish (the language of the participant of the study), and while participants were non-experts in Russian (the Cyrillic language learnt), they might have been previously exposed to it. Cyrillic characters partially overlap with Latin ones (letters A, B, I, P, C, H, X, T, are all occurring in both scripts), in some cases with similar grapheme-phoneme mapping (e.g. A, O, T), and such overlap may have posed an advantage for Cyrillic over Braille script.

To balance the novelty of the scripts, the input sensory modality, and to compare the effects of the presence or absence of line junctions in its most simple way, in our study we compare behavioural responses for the same training paradigm applied to two highly similar scripts: visual Braille and a custom script where Braille dots are connected to form line junctions. By reducing the differences between the training paradigms and between the script that are compared, we can more effectively identify whether the hypothesized advantage of lines is indeed present and, if so, to what extent.

We compared the behavioural responses of two groups of participants learning either script in the same online training setting. We observed that overall accuracy and time to provide the answer during a transcription of words from the novel to the native alphabet does not depend on which script is learnt, meaning that the visual features of the script (the only difference between them) does not pose an advantage. The only consistent but temporary advantage we observed was a faster learning in the line-based script, reflected in a significant interaction between script and session. In general, we show a limited and temporary advantage to learn a line-based script to one that does not present such features. These findings illustrate the flexibility of the visual system to adapt to peculiar stimuli that lack the typical features of objects and scripts.

## Methods

### Participants

A total of eighty participants (53 women, mean age = 22.5 ± 3.97 years) completed the experiment within the time windows requested a priori (one session per day) and were included in the analyses. An additional 21 participants were recruited but excluded prior to the start of analyses due to different reasons (14 did not complete the experiment, five did not start, and two did not complete the experiment in the required time window). Participants were assigned a script to learn at the beginning of the experiment, resulting in two matched groups which learnt either Braille (*N* = 40, 26 women, mean age = 22.52 ± 3.65 years) or Line Braille (N = 40, 27 women, mean age = 22.48 ± 4.29 years). The protocol was approved by the ethics committee at KU Leuven and all participants gave explicit informed consent prior to the start of the first training session.

### Stimuli

To compose the new alphabets, we relied on the Braille script and joined the dots forming each letter into continuous lines. For this reason, we adapted the Braille script and modified some characters. In particular, we changed the character for the letter ‘a’ from ‘⠁’ to ‘⠺ ‘ to allow for a line counterpart in in the new line alphabet; and we changed the character for the letter ‘k’ from ‘⠅’ to ‘⠡’ to avoid an overlap with the line letter ‘l’ (‘⠇’). We used letter stimuli created for a previous pilot investigation (Gastoud, 2022) in the French script. We created the Line Braille condition by joining the dots to create the shortest path possible and to avoid similarity with Latin script letters, where possible. To select the words for the training set, presented in sessions two, three, and four, we relied on the Dutch Lexicon Project 2 (Brysbaert et al., 2016). All the words we selected had high frequency (> 2/million) and word prevalence (higher than 1.65), lengths between four and eight characters and between two and six phonemes. The final set consisted of two hundred words, equally distributed based on the length of the words. From this set, we selected twenty different words for each of the three words training session (sixty words in total) to perform an interim assessment of the transcription accuracy during training. We modelled the stimulus set of the test words based on the work of Martin and colleagues (2019), where the authors presented novel words, previously seen words, and pseudowords. To choose the novel words, we adopted the same criteria used to select the training words. We chose the seen words from the training set, randomly selecting words that were not part of the interim assessment. Lastly, we choose the pseudowords from Wuggy (Keuleers & Brysbaert, 2010), a multilingual pseudoword generator. For each subset, we picked eighty stimuli of matched length, from four to eight characters. We then assigned twenty stimuli to each testing session, balancing the lengths of the stimuli across sessions.

### Experimental design

**Fig. 1:**
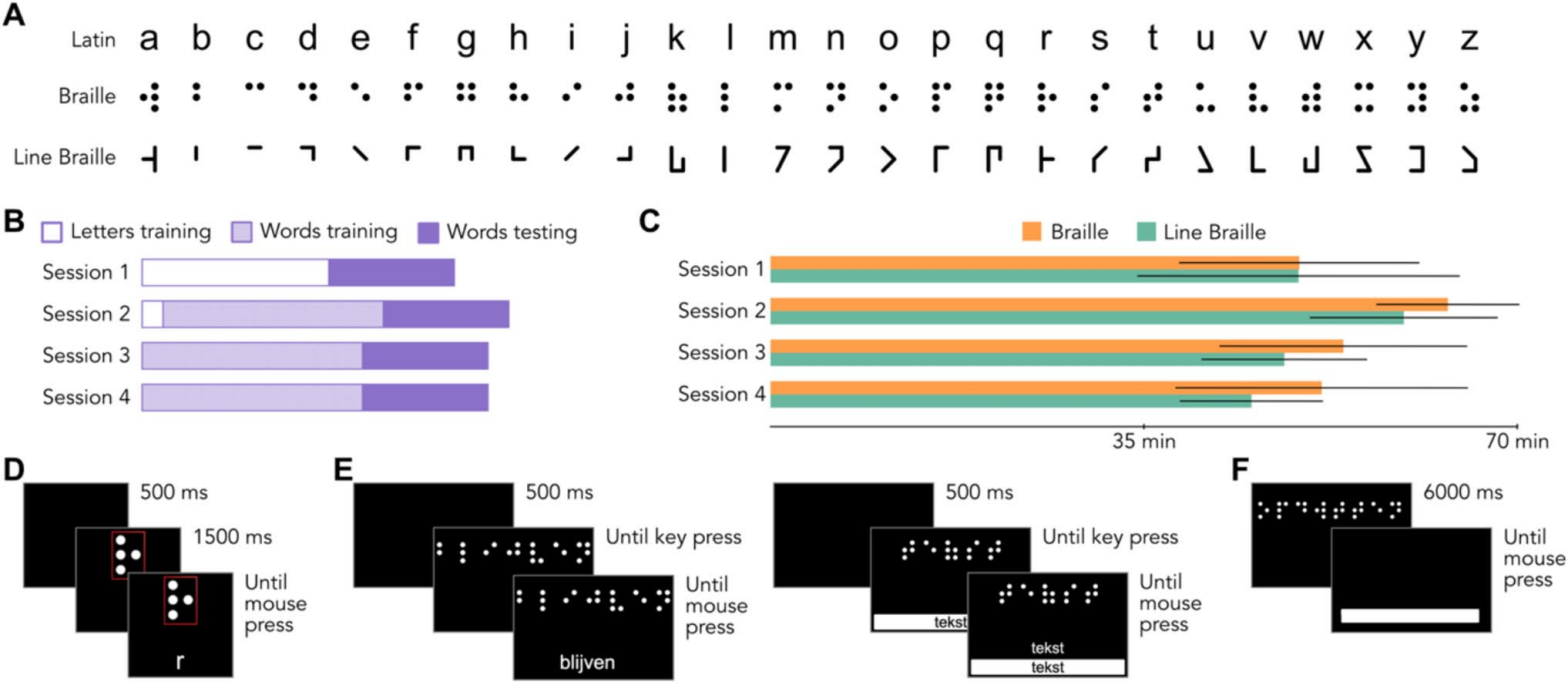
Experimental design. **A** Letters used in the experiment: Latin, Braille, Line Braille. **B** Schematic view of the training experiment. In the first session, participants saw seven cycles of letters in the new alphabet with the relative transcription and then moved to a test phase on the transcription of full words and pseudowords. In all the subsequent session, training was on full words, while testing was on words and pseudowords. At the start of the second session, participants saw another cycle of letters before starting to learn full words. **C** Average time spent in each session, divided by group. Statistical tests do not show significant differences between groups in terms of time spent on each session. **D** Trial of letters training. Each trial started with a 500-ms black screen, after which a letter in the novel alphabet was presented inside a red outline. The outline was added to provide information about the vertical position of the dots/lines. The novel letter remained on screen until participants pressed a key on their keyboard to show the corresponding Latin letter. After the transcription was presented, participants could advance via a mouse click. **E** Trials of words training. In the majority of trials (90%, left), participants were required to read the novel word presented on screen after a 500-ms interval. Only after 3,500-ms, they could show the transcription and move forward. In 10% of the trials (right), a written answer was required. Participants had to input the transcription and could check their answer by a key press. Only after the key press, they could advance to the next item. **F** Test trial. Each test trial started with a 6-s presentation of the test stimulus, after which the stimulus would disappear and an answer box would appear for the participant to input the correct transcription. The participant could then move to the next item by clicking with the mouse to submit the answer.

We presented the experiments to participants using PsychoPy (Peirce, 2007, 2008) through Pavlovia.org, a web-based platform for the presentation of experiments. With the exceptions of the script learnt and the random presentation order of the stimuli, all the participants underwent the same training procedure (Fig. 1B). All included participants performed the experiment sessions on four consecutive days. Although the experiment was self-paced, and resulted in different durations between participants, we did not find significant differences across scripts (Fig. 1C) in the duration of each session (Session 1: *t*_(78)_ = 0.02, p_Bonf_ = 1; Session 2: *t*_(78)_ = 1.16, p_Bonf_ = 0.99; Session 3: *t*_(78)_ = 1.25, p_Bonf_ = 0.86; Session 4: *t*_(78)_ = 1.38, p_Bonf_ = 0.69).

On the first session, participants had to read and accept the informed consent form to continue and start the experiment. They were then prompted to learn the new alphabet letters (Fig. 1D). Each letter was presented at the centre of the screen, surrounded by a frame to highlight the relative position of the features. After 1.5 s, the corresponding letter in the Latin script appeared. Participants could study the correspondence between scripts without a time limit, but for a minimum time of 1.5 s and could progress in the learning by a mouse press. The whole alphabet of new stimuli (26 letters) was presented for seven repetitions, with randomized letter order within one repetition. The training of letters lasted for 21 (± 7) min on average. Participants were then prompted to take a pause of approximately 5 min before starting the test, and were provided with instructions on how to perform the test.

The test phase’s methodology was identical, except for the stimuli, in each session (Fig. 1F). Participants were presented with a string of characters (a word or a pseudoword) for 6 s. We opted for a fixed presentation time of 6 s as a result of pilot tests in which we compared multiple presentation timings (6, 9, 12 s, and self-paced reading) and found a short presentation timing to be the most sensitive to script differences. After that time elapsed, the stimulus disappeared and participants had to provide a written transliteration of the stimulus in the Latin script. To submit the answer, participants had to perform a mouse press. This procedure allowed us to register not only the accuracy of the transliteration, but also the time required to provide the answer, adding a measure of learning speed, or efficiency. After an answer was submitted, participants moved to the next test item.

On the second session, participants underwent first one repetition of the letters training, to review the correspondence. This phase lasted for 2.3 (± 0.8) minutes. After the review of letters, instructions for the training experiment were provided and participant could start the session. Each item of the training set was a full word presented at the centre of the screen. In most trials, after 2.5 s participants were able to press a key to show the corresponding word in the Latin script, and only after the answer was revealed they could continue to the next trial. In the case of one item selected for the interim assessment (10% of the trials, 20 words), participants had to input a string of text to reveal the answer and to be allowed to continue the experiment (Fig. 1E). We opted to add these trials for two reasons: to obtain a measure of the pace of learning, and to ensure attentive participation in the experiment. After the training phase, which lasted on average 32.95 (± 14.36) minutes, participants were again prompted to take a break and perform another test session, in the same manner as the previous day. On the third and fourth sessions, participants performed again the words training, under the procedure but with different trials assessed, and underwent the same testing phases, with new sets of stimuli.

### Statistical analyses

To ensure that the training paradigms are comparable, we performed four independent-sample *t* tests on the durations of the sessions across scripts, and corrected for multiple comparisons using Bonferroni correction. To test whether one script was easier to learn, we extracted several measures of accuracy and timing from the results of the three sessions on words training and the four test sessions. From the test results, we computed the accuracy for each participant at each session. We also computed the writing time, the time required to provide the written answer. We measured such time by the time between the first key press and the last key press on the keyboard. In this computation, we considered as an outlier any case where the total time was higher than 60 s, or when the time between two key presses was higher than 30 s. We chose this measure to exclude potential outlier cases where the participant, to pause the experiment, would leave the completed answer on screen before moving to the next item. We additionally computed accuracy during the training interim assessment and the time required to process the words not assessed. In this case, we considered as outlier any trial that required more than 60 s, for the same reason described above.

For each behavioural measure, we performed repeated measures ANOVA on the test (2 scripts × 4 test sessions) and on the training (2 scripts × 3 training sessions) results. When an interaction effect was found, we performed post hoc tests by means of four *t* tests on independent samples on the differences between scripts at each session. We corrected those test for multiple comparisons using Bonferroni correction. To further interpret whether the lack of significant effect corresponds to a lack of finding or to the finding of a lack of effect, we computed Bayes factor analyses (Kass & Raftery, 1995). We additionally report the confidence interval of the difference in means (95%) in the cases of nonsignificant *t* tests. To investigate the role played by the linguistic properties of the stimuli learnt, we computed correlations between the results from each participant and the stimuli’s length, frequency, and orthographic neighbours. To identify significant correlations between behaviour and linguistic properties, we performed, for each behaviour-linguistic correlation, a one-sample *t* test against chance (zero) on the average correlation per participant. We then performed repeated measures ANOVAs on the individual correlations to examine differences between scripts and sessions. For all the repeated measures ANOVAs, we estimated effect size through partial eta-squared (η_p_^2^).

## Results

### Transcription accuracy during testing

**Fig. 2:**
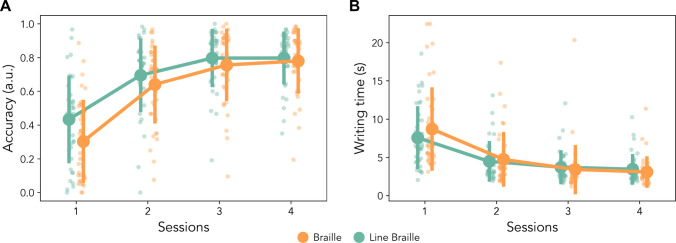
Accuracy and time to write the transliteration during test. **A** Accuracy in the transcriptions. A main effect of session indicates that the transcriptions improve over time, while the lack of group differences indicates that the two scripts are learnt in a similar, comparable manner. There was a significant interaction between session and script. **B** Writing time to provide the transcription. The speed of transcription improves during the training, without differences between the scripts, nor interactions between scripts and sessions. Error bars represent standard error

To assess whether the two scripts can be transcribed from the novel to the Latin alphabet in a similar manner, the first and most straightforward measure we obtained was transcription accuracy (Fig. 2A). Overall, across the four testing sessions, we observed a main effect of session (*F*_(3, 234)_ = 236.231,* p* < 0.001, η_p_^2^ = 0.75), indicating that the participants in both groups improve over time. We did not observe a main effect of the script tested (*F*_(1, 78)_ = 2.312,* p* = 0.13, η_p_^2^ = 0.03). This lack of significance shows that, as early as after a few hours of training divided in four consecutive days, the two scripts reach comparable levels of accuracy and are therefore assimilated in a similar manner. Interestingly, we do observe a significant interaction between sessions and scripts (*F*_(3, 234)_ = 3.678, *p* = 0.012, η_p_^2^ = 0.05), which highlights a difference between the learning curves. To clarify the nature of such interaction, we performed post-hoc analyses on each session to identify where a difference between script could be present. We observed a difference only in the first session (*t*_(78)_ =  − 2.39, p_uncorr_ = 0.018, 95% CI [− 0.24, − 0.02]) and not in the following ones (Sessions 2, 3, 4: 95% CIs [− 0.15, 0.04], [− 0.12, 0.04], [− 0–09, 0.06]; all p_uncorr_ > 0.26), supporting the interaction and the lack of main differences between scripts. However, no significant test survives Bonferroni correction (Session 1: p_Bonf_ = 0.076; Sessions 2, 3, 4: all p_Bonf_ = 1). Such limited interaction is supported by Bayes factors, which show an initial limited evidence (Session 1: BF = 2.69, err. < 0.001) for the difference between scripts, but increasing evidence for the null hypothesis in the following sessions (Session 2: BF = 0.41, err. = 0.0015; Session 3: BF = 0.35, err. = 0.0016; Session 4: BF = 0.26, err. = 0.0017).

### Writing time during word testing

Similarly to accuracy, we investigated the time required to transcribe the (pseudo)words presented into their native Latin script (Fig. 2B). We performed a repeated-measures ANOVA (rmANOVA) on sessions × script and found only a main effect of session (*F*_(3, 234)_ = 98.451, *p* < 0.001, η_p_^2^ = 0.56), indicating a progressive improvement and efficiency in the visual learning task. We did not find significant differences between script (*F*_(1, 78)_ = 0.099, *p* = 0.75, η_p_^2^ = 0.001) nor interaction effects between the variables (*F*_*(*3, 234)_ = 2.334, *p* = 0.075, η_p_^2^ = 0.03). Overall, the learning progression seem to be equal between the two scripts as far as it can be assessed through the time that participants take to write the words in full. Although not significant, we explored the interaction trend between scripts and sessions. Post hoc *t* tests on the script difference reveal no significant differences at any point (95% CIs: Session 1 [− 0.92, 3.19], Session 2 [1.05, 1.57], Session 3 [− 1.44, 0.83], Session 4 [− 1.16, 0.46]; all sessions p_uncorr_ > 0.28, p_Bonf_ = 1), with Bayes factors pointing in favour of the null hypothesis (Session 1: BF = 0.39, err. < 0.001; Session 2: BF = 0.24, err. < 0.001; Session 3: BF = 0.26, err. < 0.001; Session 4: BF = 0.32, err. < 0.001).

### Influences of language statistics

**Fig. 3:**
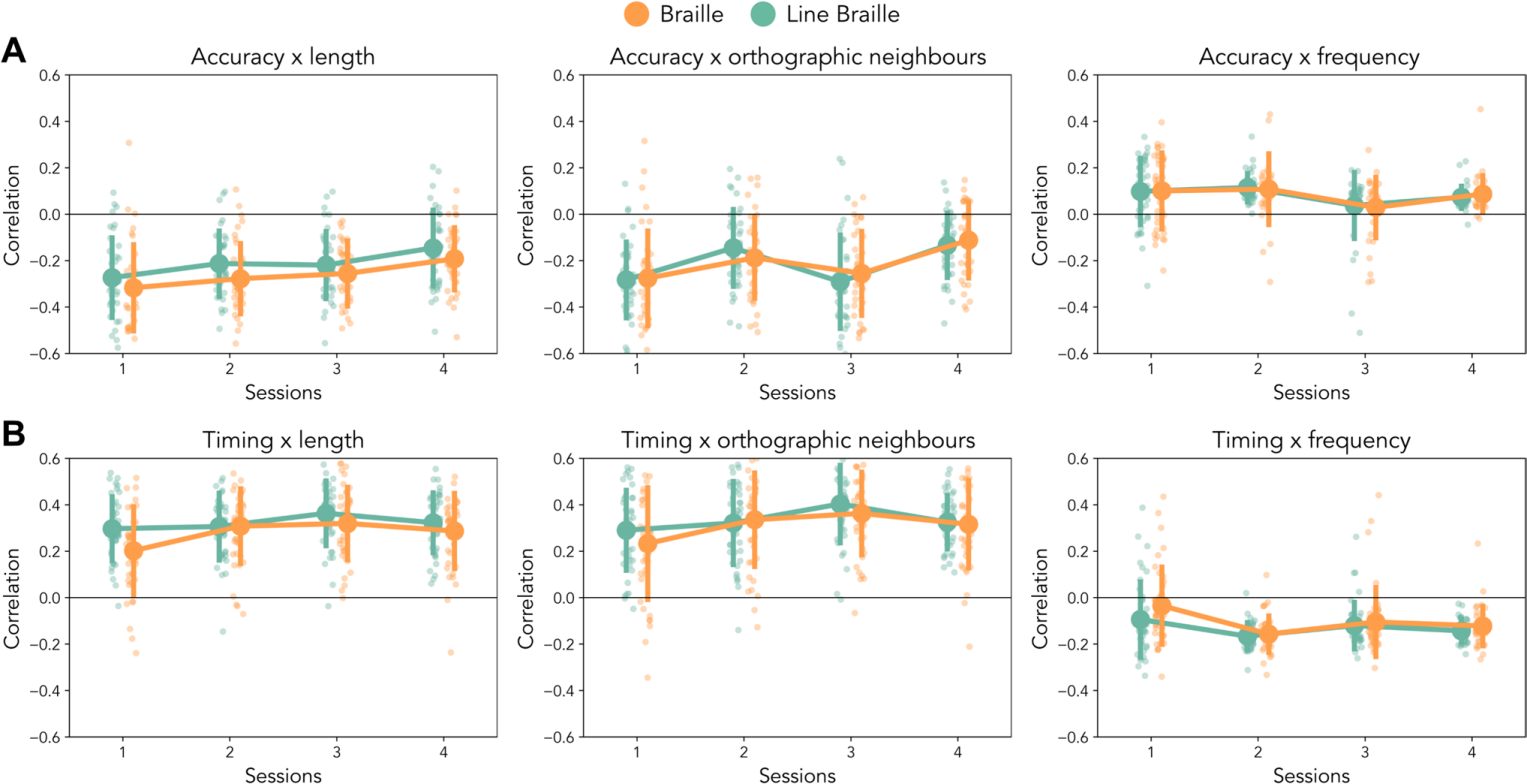
Correlations between behavioural variables and language statistics. **A** Correlations between accuracy during test and stimulus length (left) show more sensitivity in transcription from Braille than from Line Braille, indicating a lower assimilation of the linguistic components. Words’ orthographic neighbours (centre), and frequency (right) do not show differences between groups. All variables show a session effect which highlights lower errors with time. **B** Correlations between writing time during test and stimulus length (left), words’ orthographic neighbours (centre), and frequency (right) all show only main effects of the session and no group differences. Error bars represent standard error

To assess whether the differences observed were influenced by statistics of the stimuli presented, we performed correlations between the accuracy measures and writing time with the linguistic statistics of the word and pseudowords used in the test sets (Fig. 3). To extract language statistics of the novel and seen words subsets, we relied on the Dutch Lexicon Project (Brysbaert et al., 2016) to extract information about the length, the frequency, and the orthographic neighbours (old20) of a given word. For the pseudowords, we could only compute a measure of the length of the word, therefore the correlations with length will refer to the whole corpus of test stimuli presented (*N* = 60 per session), while correlations with frequency and orthographic neighbours will be limited to the subset of real words (*N* = 40 per session).

We observe, in all the cases, overall significant correlations between the behavioural measurements and the linguistic statistics of the stimuli. Accuracy correlates negatively with the length of the stimulus, with less accurate responses for longer stimuli (*r* =  − 0.23, *t*_(79)_ =  − 21.30, p_Bonf_ < 0.001) and with orthographic neighbours of the target words (*r* =  − 0.21, *t*_(78)_ =  − 19.42, p_Bonf_ < 0.001), meaning that words with more orthographic neighbours lead to more errors, but positively with word frequency (*r* = 0.08, *t*_(78)_ = 12.02, p_Bonf_ < 0.001), which indicates that more frequent words are easier to transcribe than nonfrequent ones. As for the time to write the answers, we observe opposite trends (timing-length: *r* = 0.30, *t*_(79)_ = 25.58, p_Bonf_ < 0.001; timing-ort. neighbours: *r* = 0.32, *t*_(79)_ = 25.69, p_Bonf_ < 0.001; timing-frequency: *r* =  − 0.12, *t*_(79)_ =  − 17.42, p_Bonf_ < 0.001) but with similar interpretations: longer stimuli induce longer writing time, and words with more similar orthographic neighbours lead to more time to complete the word. Lastly, more frequent words require less time to write.

More in depth, we compared the influence of each linguistic statistic over the progression of the two groups across test sessions. Regarding the correlation between transcription accuracy and stimulus length, we found a significant effect of session (*F*_(3, 216)_ = 8.52, *p* < 0.001, η_p_^2^ = 0.11) and a difference between the scripts across the whole training (*F*_(1, 72)_ = 6.06, *p* = 0.016, η_p_^2^ = 0.078). Such difference and its effect size indicate that participants who learnt Braille were slightly more sensitive to the length of the word or pseudoword presented (i.e., more prone to errors if the word was longer) than the group who learnt Line Braille. No interaction is present between script and session (*F*_(3, 216)_ = 0.22, *p* = 0.88, η_p_^2^ = 0.003).

In the correlations between accuracy and orthographic neighbours (session: *F*_(3, 189)_ = 14.13, *p* < 0.001, η_p_^2^ = 0.97; script: *F*_(1, 63)_ = 0.65, *p* = 0.42, η_p_^2^ = 0.01; script × session: *F*_(3, 189)_ = 1.00, p = 0.39, η_p_^2^ = 0.02), and between accuracy and word frequency (session: *F*_(3, 189)_ = 4.21, *p* = 0.006, η_p_^2^ = 0.06; script: *F*_(1, 63)_ = 0.04, *p* = 0.83, η_p_^2^ < 0.001; script × session: *F*_(3, 189)_ = 0.11,* p* = 0.95, η_p_^2^ = 0.001) we only observe main effects of session and no script differences, which indicate similar progression in learning across days of training. Between writing time and linguistic statistics (Fig. 3B), we found significant main effects of the training sessions and no difference between scripts. This is valid for the correlations between writing time and stimulus length (session: *F*_(3, 234)_ = 6.78,* p* < 0.001, η_p_^2^ = 0.08; script: *F*_(1, 78)_ = 3.47, *p* = 0.07, η_p_^2^ = 0.04; script × session: *F*_(3, 234)_ = 1.86, *p* = 0.13, η_p_^2^ = 0.02), word orthographic neighbours (session: *F*_(3, 234)_ = 7.13,* p* < 0.001, η_p_^2^ = 0.08; script: *F*_(1, 78)_ = 0.85, *p* = 0.36, η_p_^2^ = 0.01; script*session: *F*_(3, 234)_ = 0.73, *p* = 0.53, η_p_^2^ = 0.01), and word frequency (session: *F*_(3, 234)_ = 10.40, *p* < 0.001, η_p_^2^ = 0.11; script: *F*_(1, 78)_ = 3.86, *p* = 0.053, η_p_^2^ = 0.05; script × session: *F*_(3, 234)_ = 0.80, *p* = 0.49, η_p_^2^ = 0.01).

### Transcription accuracy and reading timing in word training

**Fig. 4:**
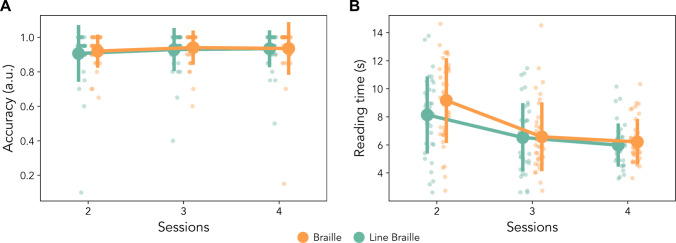
Accuracy and reading time during training. **A** Accuracy in the transcriptions during the training. No significant result emerged from the analysis, the performance is already at ceiling in both groups from the first training session on words. **B** Time spent reading the words in the novel alphabet. Participants need less time to process the word when training progresses. Error bars represent standard error

To explore further possible differences between the two scripts, we further measured the accuracy transcription, and reading time during the training on words presented in sessions two, three, and four (Fig. 4). For transcription accuracy, we measured accuracy on the 10% of training trials that required a written answer (*N* = 20). We did not find any effect of script (*F*_(1, 78)_ = 0.15, *p* = 0.69, η_p_^2^ = 0.001), session (*F*_(3, 156)_ = 2.275, *p* = 0.11, η_p_^2^ = 0.03), nor interaction (*F*_(3, 156)_ = 0.165, *p* = 0.85, η_p_^2^ = 0.002). This lack of effect might be caused by the limited number of items tested in this phase, combined with the ceiling performance.

Reading time was defined as the amount of time passed between the start of the trial and the time participants pressed the key to visualize the correct transliteration, and could be assessed on the remaining 180 trials for each training session. In this case, we observe a main effect of session (*F*_(3, 156)_ = 81.347, *p* < 0.001, η_p_^2^ = 051) associated to the progression during the whole experiment. Moreover, we do not observe any significant difference between scripts (*F*_(1, 78)_ = 0.975, *p* = 0.33, η_p_^2^ = 0.01) and a marginally significant interaction (*F*_(3, 156)_ = 2.967, *p* = 0.054, η_p_^2^ = 0.04). The trend of the interaction, albeit not significant, is consistent with the interaction found on accuracy during testing, with a tendency towards a difference between scripts early in training (here: slower reading time for Braille) and no sign of any difference in the last session. More in depth, the post hoc *t* tests do not highlight any significant script difference during any session (Session 2: *t*_(78)_ = 1.66, p_uncorr_ = 0.10, p_Bonf_ = 0.31, BF = 0.76, err. < 0.001, 95% CI [− 0.21, 2.26]; Session 3: *t*_(78)_ = 0.09, p_uncorr_ = 0.93, p_Bonf_ = 1, BF = 0.23, err. < 0.001, 95% CI [− 0.99, 1.08]; Session 4: *t*_(78)_ = 0.70, p_uncorr_ = 0.48, p_Bonf_ = 1, BF = 0.29, err. < 0.001, 95% CI [− 0.42, 0.88]).

## Discussion

We conducted a four-days online behavioural training where participants learnt to read either visual Braille or a custom script with dots connected into lines. Overall, we showed that the accuracy at a transcription test improves over time in a similar fashion between scripts. There was a small advantage for the line Braille in the speed of learning, reflected in a significant interaction between script and session. Writing time during test improved over learning, but did not differ between scripts. Linguistic factors, including word length, number of orthographic neighbours, and frequency, affected task accuracy and writing time, but mostly did so in a similar way in the two scripts. Overall, we show comparable learning trajectories between Braille and a line-based script, hinting at a limited role of the presence of lines and junctions in scripts, except for a small and temporary effect of the presence/absence of lines on the speed of learning.

The interaction shows that transcription from Line Braille is more accurate than from Braille with dots earlier in training, revealing at best a temporary advantage of the presence of lines. Additionally, we also see an overall stronger correlation between stimulus length and Braille script compared to the line-based one. Such higher sensibility to length could also be a proxy of a slightly weaker mapping between the novel and the native alphabet. Together, this evidence could lead to the conclusion that some line advantage is indeed present. After all, the two scripts are visually very different, and no visual script evolved naturally to be represented by dots (Changizi et al., 2006). It remains possible that transcriptions from Braille are more prone to errors, reflecting a less strong mapping. The differences between scripts are temporary and towards the end of four days of training there is no difference in accuracy during test, nor in reading speed during training. While we only found a temporary advantage for learning Line Braille, It is possible that such a temporary benefit of a line-based alphabet would still be meaningful in real-world literacy acquisition and would have long-term consequences for reading fluency with longer exposure to those scripts. This could be explored in longer longitudinal studies.

The conclusion that the scripts converge to similar assimilation towards the end of training is further complemented by the Bayes factors, which show moderate evidence in favour of the null hypothesis in the last training session. However, given limitations of Bayes factors in the sample size (*Nb* = 40 for each group) and in the definition of the prior assumptions (a general lack of difference), extending these findings to other measures of literacy could provide additional information. We measured similar learning accuracy of the novel scripts, regardless its presence or absence of line-based visual features. Previous literature hypothesised an advantage of line-junctions processing when reading that builds over the already present circuitry for object recognition (Dehaene & Cohen, 2011; Dehaene et al., 2005). The importance of line-junctions as building blocks of a visual script suggested hindered processing when such features were absent (Szwed et al., 2009, 2011). Moreover, while many conducted training experiments on peculiar scripts, derived from example from other categories found to activate areas in vOTC, like houses and faces (Martin et al., 2019; Moore et al., 2014), those studies looked at more complex stimuli that potentially recruit the same organizational processes of line-junctions integration. Rarely there has been a direct comparison between scripts with different organizational principles, line-junctions integration versus dots patterns. Bola and colleagues (2017) did so and compared learning visual Braille to Cyrillic. The authors show how learning Cyrillic, being made out of canonical line-junctions, poses an advantage to achieve fluent reading. However, differences remained between training programs. In our study, we balanced such conditions: participants undergo the same training regime, with consistent timings and sessions across groups; participants see equally novel stimuli that rarely have been seen outside the experimental setting and the same transcribed words in their native script (Latin). We show that when the trainings are balanced, the advantage of line-junctions is only very temporary and disappears with training.

We also explored whether one script would be more sensitive to the linguistic properties, namely stimulus length, word frequency, and orthographic neighbours of the stimuli presented. These linguistic factors influenced task performance in obvious ways, with for example correlations between accuracy and writing time at the one hand and word length and the number of orthographic neighbours at the other hand. However, the effects of these factors almost never differed between the two scripts, corroborating the overall similarities in learning a new alphabet independently of the presence of lines and line junctions.

One limitation of our testing protocol is that the stimuli were always presented for 6 s. With this protocol we might miss effects in speed of reading. Yet this does not seem to be the case if we take the results as a whole. First, maximum accuracy at test is 80%, which is lower than the ceiling and lower than accuracy during training when there is no time limit (the latter shown in Fig. 4A). This suggests that presentation time during the test phase is brief enough to avoid ceiling effects, and warrants evaluating group differences. Second, even during training where presentation duration is self-paced, there are no indications of differences in the processing speed during the later sessions (Fig. 4B). Overall, and also considering the reported Bayes factors, our findings indicate that the Braille with dots and the Line Braille with connected dots are associated with equal accuracy and speed in the later training and test sessions. It remains possible that more challenging tasks, or a more direct measure of reading fluency, could highlight more marked differences between scripts and different advantages due to the presence or absence of line junctions.

In our study, we compared two scripts on their (lack of) apparent lines and we did not address the possibility that participants might perceive imaginary connecting lines between Braille dots. It remains possible that dots are completed into lines, following studies on the perception of illusory contours (Kanisza, 1976; Kennedy & Ware, 1978). To answer this question, we warrant the need for further studies focused on the conditions needed for such illusion to emerge (variating the sizes of dots and the distance between them could pinpoint which conditions determine illusory perception) and on the neural activations related to conditions of dots, of dots joined into lines, of lines imagined between dots. Such studies could identify whether the dots are merged into lines and what is the resulting encoding of information.

**Fig. 5:**
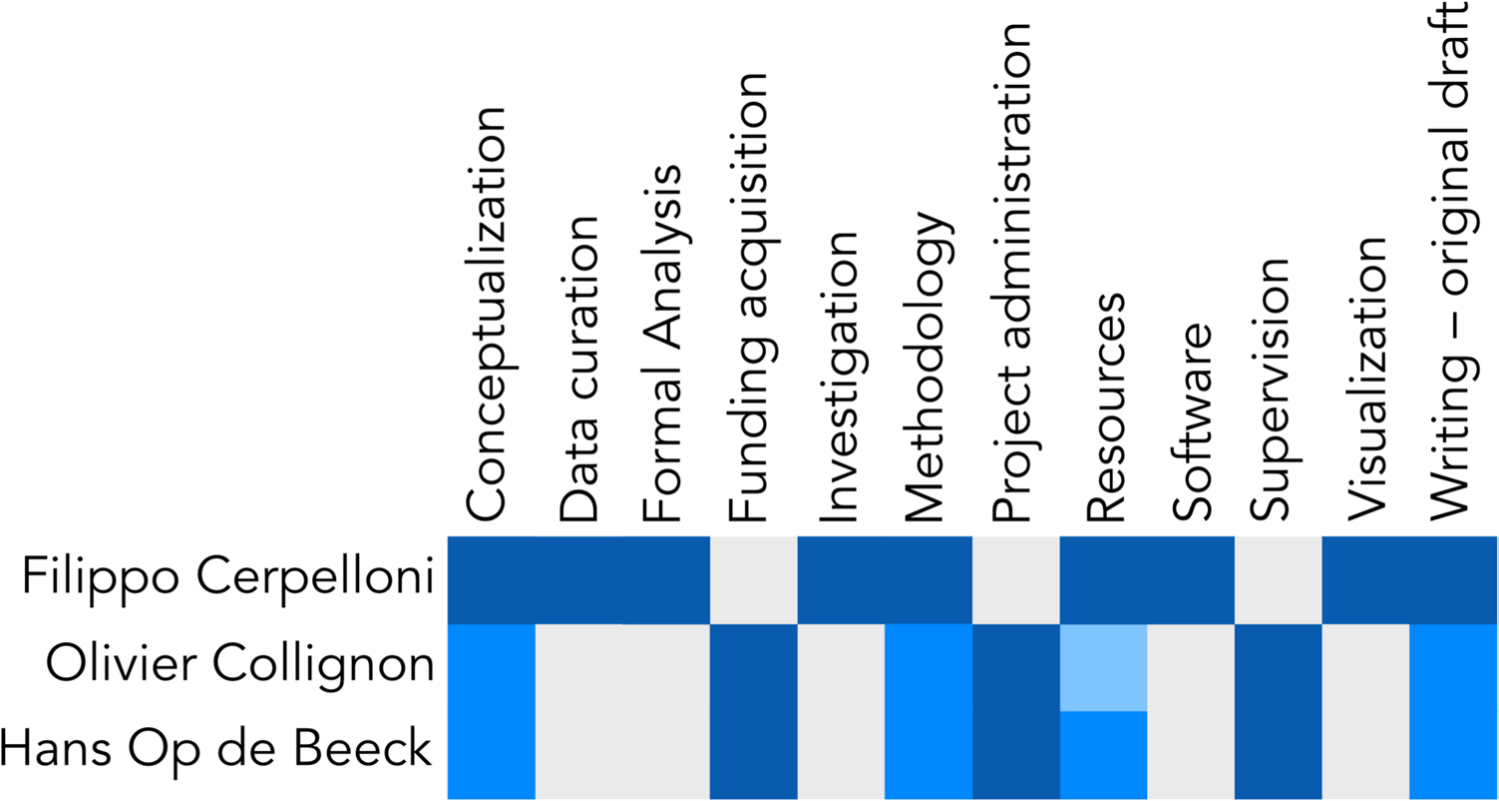
Author contributions. Based on the CRedIT (https://credit.niso.org/) taxonomy. Each contribution was assessed as ‘lead’ (dark blue), ‘equal’ (mid blue), ‘support’ (light blue)

The proficiency of participants to learn the visual Braille with dots and the effect of factors like orthographic neighbours are consistent with our previous observations that expertise in visual Braille engages the same reading network and neural coding principles as line-based scripts. We previously observed that extensive training in visual Braille leads to similar activity across the reading brain network when compared to the ones observed in the participants’ native Latin alphabet (Cerpelloni et al., 2025). Given the malleability of the reading network to adapt to various scripts (Baker et al., 2007; Martin et al., 2019; Moore et al., 2014; Xue & Poldrack, 2007) and in particular to tactile and visual Braille (Cerpelloni et al., 2025; Reich et al., 2011; Siuda-Krzywicka et al., 2016), we hypothesize that our training procedure rely on such adaptability to trigger equal performance across script types.

All this evidence could be seen in favour of concurrent theories about reading in the brain, which stress the importance of the linguistic content of the stimuli rather than their visual features. Under this assumption, reading could be seen as a mapping of symbols to meaning, a mapping not restricted by the visual features of the stimuli.

In conclusion, we show that the presence or absence of line-junctions in a novel script, under balanced training conditions, is only associated with a temporary difference in the speed of learning but no differences in later training phases, illustrating the flexibility of the human visual system and reading network to adapt to a wide range script structure.

## Data Availability

Data and materials are available on GitHub: experiments (https://github.com/fcerpe/VBT_experiments) and analyses (https://github.com/fcerpe/VBT_data).

## Code availability

All code to administer the different training sessions (https://github.com/fcerpe/VBT_experiments) and to analyse the results (https://github.com/fcerpe/VBT_data) is publicly available on GitHub.
